# Identification and genome characterization of novel parechovirus sequences from *Hipposideros armiger* in China

**DOI:** 10.1186/s12985-022-01806-1

**Published:** 2022-05-15

**Authors:** Jian Zeng, Zijun Yang, Wentao Guo, Xiaochun Wang, Shixing Yang, Quan Shen, Hao Wang, Wen Zhang

**Affiliations:** 1 Department of Microbiology, School of Medicine, Jiangsu University, Zhenjiang, 212013 Jiangsu China; 2 Qinghai Institute of Endemic Disease Prevention and Control, Xining, 811602 Qinghai China; 3 Department of Clinical Laboratory, The Affiliated Huai’an Hospital of Xuzhou Medical University, Huai’an, 223002 Jiangsu China

**Keywords:** Parechoviruses, Virome of bats, Metagenomic analysis, Yunnan province, *Hipposideros armiger*

## Abstract

**Background:**

Bats were identified as a natural reservoir of emerging and re-emerging infectious pathogens threatening human health and life.

**Methods:**

This study collected 21 fecal samples of *Hipposideros armiger* in Mengla County of Xishuangbanna Prefecture Yunnan Province to combine one pool for viral metagenomic sequencing.

**Results:**

Two nearly complete genomes of parechoviruses, BPeV11 and BPeV20, were sequenced. Genome analysis revealed that BPeV11 and BPeV20 follow a 3-3-4 genome layout: 5′ UTR-VP0-VP3-VP1-2A-2B-2C-3A-3B-3C-3D-3′ UTR. The prevalence of BPev11 and BPev20 by Nested-PCR showed that 1 of 21 fecal samples was positive. Based on amino acid identity comparison and phylogenetic analysis of P1, 2C, and 3D, BPeV11 and BPeV20 were closely related to but distinct from FPeVs.

**Conclusion:**

It was probably proposed to be a novel species in the genus *Parechovirus* of the family *Picornaviridae*. The isolation of BPev11 and BPev20 from *H. armiger* in China is the first complete genome of parechovirus isolations from bat feces of the genus *Hipposideros*.

**Supplementary Information:**

The online version contains supplementary material available at 10.1186/s12985-022-01806-1.

## Background

The *Picornaviridae* is a family of viruses with single-stranded, highly diverse positive-sense, non-segmented RNA genomes with a poly(A) tail. The family contains > 30 genera and > 75 species, but many viruses are presently awaiting classification. Picornaviruses may cause subclinical infections of humans and animals or conditions ranging from inapparent or mild febrile illness to severe heart, liver, and central nervous system diseases [1, 2]. *Parechovirus*, a genus of the family *Picornaviridae*, was recently classified into six species: *Parechovirus A*, *Parechovirus B*, *Parechovirus C*, *Parechovirus D*, *Parechovirus E*, and *Parechovirus F*. *Parechovirus A,* including 19 genotypes (HPeV-1 to -19) of Human parechovirus (HPeV) [3], is only a species of *Parechovirus* genus which can cause various human diseases ranging from asymptomatic or mild gastrointestinal and respiratory illness to severe infections involving the central nervous system[4–6]. *Parechovirus B* includes 6 genotypes of Ljungan virus ((rodent host). Ljungan virus has been associated with the aetiological agent of myocarditis, diabetes, and possibly other human diseases [7, 8]. *Parechovirus C* [9] (formerly Sebokele virus 1) contains only one genotype. *Parechovirus D* [10] (ferret parechovirus), *Parechovirus E* [11] (falcon parechovirus), and *Parechovirus F* [12] (gecko parechovirus) are the same as *Parechovirus C*. The knowledge of picornaviruses host range, geographical distribution and genome organization has recently exploded due to the use of high-throughput sequencing and the identification of novel picornaviruses from various species [10].

Bats, the only flying mammal and account for more than 20% of the subsistent mammals, were recently identified as a natural reservoir of emerging and re-emerging infectious pathogens [13], many of which could spillover into animal and human populations, such as severe acute respiratory syndrome coronavirus (SARS-CoV), Middle East respiratory syndrome coronavirus (MERS-CoV), coronaviral disease-19, Nipah virus, Hendra virus, and Ebola virus [14, 15]. Hence, investigating viruses in bats is critical for improved control and prevention of large epidemics.

In this study, two nearly complete genomes of parechoviruses were sequenced and analyzed from the fecal samples of *Hipposideros armiger* in Mengla County of Xishuangbanna Prefecture Yunan Province by metagenomic analysis.

## Methods

### Sample collection and pool preparation

**Fig. 1:**
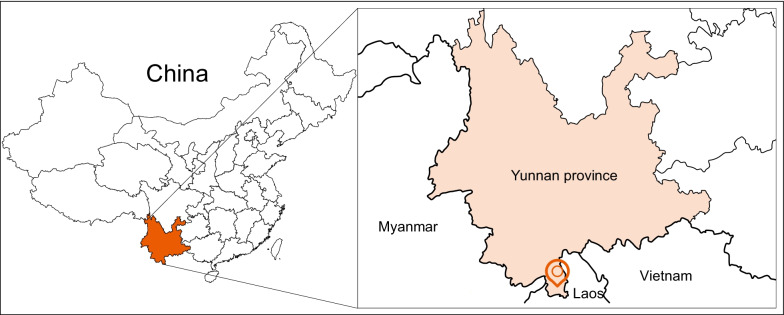
Map of the Yunnan province of China showing sampling locations for the current study. Orange coordinates show Mengla County of Xishuangbanna Prefecture Yunnan Province. The county names are shown

During 2017, a total of 21 fresh fecal samples were collected from wild *H. armiger*. The study was conducted in Mengla County of Xishuangbanna Prefecture Yunnan province, which is connected with Laos in the east and south and faces Myanmar across lancang River in the west, as shown in Fig. 1. All samples were collected with disposable materials, shipped on dry ice and stored at − 80 °C for further study. The collected fecal samples were mixed into a group, suspended in 600 µl of Dulbecco's phosphate-buffered saline (DPBS), and then vigorously vortex oscillation for 5 min. The 500 µl supernatants were then collected from each pool after centrifugation (5 min, 15,000 g, 4 °C).

### Viral metagenomic analysis

The 500 µl supernatant was purified through a 0.45-μm filter (Millipore) to remove eukaryotic and bacterial cell-sized particles. The filtrate was treated for 60 min at 37 °C with a DNases mixture (Turbo DNase from Ambion, Baseline-ZERO from Epicentre), benzonase (Novagen), and RNase (Fermentas) to digest unprotected nucleic acid. Nucleic acids (total DNA and RNA) were then extracted using a QIAamp Viral RNA Mini Kit (QIAGEN) following the manufacturer’s instructions. Total nucleic acids were subjected to RT reactions with SuperScript III reverse transcriptase (Invitrogen), following second-strand cDNA synthesis with Large (Klenow) fragment (NEB). The library was then constructed using Nextera XT DNA Sample Preparation Kit (Illumina) and sequenced using the MiSeq Illumina platform with 250 base pair-ends with dual barcoding.

### Bioinformatics analysis

Paired-end reads of 250 bp generated by MiSeq sequencing were debarcoded using vendor software from Illumina. An in-house analysis pipeline running on a 32-node Linux cluster was used to process the data. Reads were considered duplicates if bases 5 to 55 were identical and only one random copy of duplicates was kept. Clonal reads were removed, and low-sequencing-quality tails were trimmed using Phred. Adaptors were trimmed using VecScreen with the default parameters, which uses NCBI BLASTn with specific parameters designed for adapter removal. The cleaned reads were then compared to an in-house non-virus non-redundant (NVNR) protein database to remove false-positive viral hits using DIAMOND BLASTx search with default parameters [16]. The NVNR database was compiled using non-viral protein sequences extracted from an NCBI nr fasta file (based on annotation taxonomy, excluding the virus kingdom). Then, taxonomic classification for DIAMOND results was parsed using MEGAN to perform the LCA-assignment algorithm according to default parameters. Gene assembly, prediction, and annotation were completed with Geneious software [17].

### Nested PCR

Nested PCR was performed using rTaq DNA Polymerase (Takara) to amplify complete DNA or RNA and determine whether exist the target viruses. The specific primer sequences are shown in Table 1.

**Table 1:** Specific gene primers used in nested PCR

Virus	Application	PrimerID	Primer sequence (5′–3′)	Product length (bp)
BPeV11	First round	WF	GCGGTCTTCCAAACCAAACC	623
		WR	CTGGCAAAGTCACCAAGTGC	
	Second round	NF	TGCTTGGCTTGGAGACAGAG	316
		NR	ACACATGACCCCCGGATAGA	
BPeV20	First round	WF	AGAACCTGCAGTGCTCTCAC	604
		WR	GGGGAAAAGACTACGCACCA	
	Second round	NF	GGCTGCTGTCAACAATGTGG	335
		NR	ATGCCTCAATGCACCTGGTT	

### Phylogenetic analysis

The predicted potential proteins were aligned with their corresponding homologs of reference viruses using the MUSCLE multiple sequence alignment program with default settings [18]. The RdRp is the only conserved-sequence domain across all RNA viruses and was used for phylogenetic inference [19]. All phylogenetic analysis was performed based on a Bayesian method implemented in MrBayes version 3.2.7 [20, 21]. In the MrBayes analyses, we used two simultaneous runs of Markov chain Monte Carlo sampling, and the runs were terminated upon convergence (standard deviation of the split frequencies < 0.01) [22]. The visualization and beautification of the phylogenetic trees were achieved by Figtree version 1.4.4 (available from http://tree.bio.ed.ac.uk/software/figtree/).

### Prediction of protein domains and functions

**Fig. 2:**
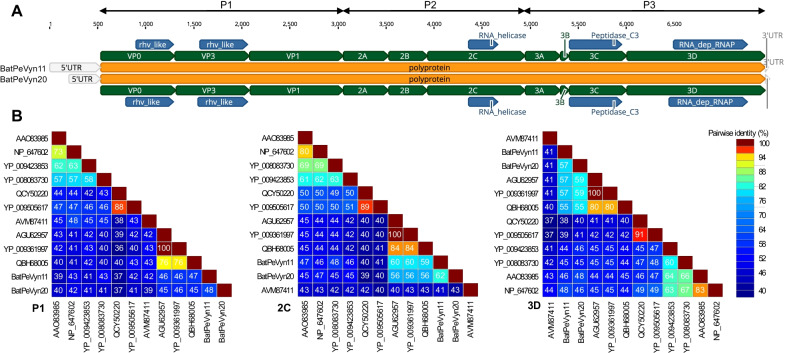
Genomic characterization and sequences identity matrices of BPeV11 and BPeV20. **A** Predicted genome organization of BPeV11 and BPeV20. P1 represents viral structural proteins, and P2 and P3 represent non-structural proteins. Positions of predicted cleavage sites are indicated along the polyprotein (green). Conserved picornaviral amino acid domains are presented with the blue bar. **B** Sequence identity matrices are shown below for P1, 2C, and 3D regions of BPeV11 and BPeV20 compared with viruses within genus *Parechovirus*

All protein prediction was conducted by Geneious prime version 2019.2.3 [19]. The conserved domains were determined using the NCBI conserved domain search in combination with the Pfam conserved domain search [23, 24]. The cleavage sites of the BPev11 and BPev20 polyproteins were predicted by sequence alignment comparisons to the polyproteins of other viruses within the genus *Parechovirus* (See Additional file 1) using Geneious prime version 2019.2.3. The same approach was implemented in order to predict the polyprotein cleavage sites of three other closely related fish picornaviruses (Wenling bighead beaked sandfish picornavirus, Guangdong spotted longbarbel catfish picornavirus, West African lungfish picornavirus, and clownfish picornavirus) that had not previously been annotated [12, 25]. Pairwise genetic comparisons of the aa sequences of the P1, 2C, and 3D regions of BPev11 and BPev20 polyprotein were compared to those parechoviruses (Fig. 2B) using the Sequence Demarcation Tool v1.2 [26], with the MUSCLE alignment option implemented.

## Result

### Identification and prevalence of novel parechovirus sequences

The 21 fecal samples of *H. armiger* in the Yunnan Province were combined into one pool for viral metagenomic sequencing. The Illumina MiSeq outputted a total of 581,026 of 250-base pair-end reads and then removed duplicate, low-quality, and cellular reads. After de novo assembly, we acquired two contigs that be similar to parechovirus. Then, the two novel parechovirus sequences, BatPeVyn11 (BPeV11) and BatPeVyn20 (BPeV20) were identified by reads mapping. Nested-PCR was employed to investigate the prevalence of BPev11 and BPev20 using a primer pair. Amplicons of approximately 316 and 330 base pairs were obtained from 2 of 21 fecal samples (sample 11 and 20), respectively. The primer sequences are shown in Table 1.

### Genomic characterization of BPeV11 and BPeV20

The nearly complete genomic sequence of BPeV11 is 7143 nucleotides (nt) in length, which is within the range found for other picornaviruses (6938–9035 nt). A single ORF (nt 522-7142) was predicted to encode a polyprotein precursor of 2207 aa, which can be artificially divided into three parts: P1, P2, and P3. Similarly, the nearly complete 7096-nt-long RNA genome of BPeV20 has a single ORF (nt 320 -7048), which encodes a polyprotein precursor of 2243 aa (Fig. 2A). BPev11 and BPev20 both follow a 3-3-4 genome layout: 5′ UTR-VP0-VP3-VP1-2A-2B-2C-3A-3B-3C-3D-3′ UTR (Fig. 2A). The Predicted cleavage sites for genes of BPev11 and BPev20 are shown in Fig. 2A and Table 1, based on a Muscle-alignment with fourteen previously annotated parechoviruses showing cleavage sites. In the polyprotein of BPeV11 and BPeV20, five conserved domains could be identified by an NCBI combined with a Pfam conserved domain search (Fig. 2A). The specific sites and lengths are listed in Table 2.

**Table 2:** Predicted genome organization of BPev11 and BPev20

Name	Type	BPeV11				BPeV20			
		Nucleotide			Protein length	Nucleotide			Protein length
		Minimum	Maximum	Length		Minimum	Maximum	Length	
5'UTR	5'UTR	1	521	521		1	319	319	
polyprotein	ORF	522	7142	6621	2207	320	7048	6729	2243
VP0	mat_peptide	522	1253	732	244	320	1072	753	251
VP3	mat_peptide	1254	1991	738	246	1073	1798	726	242
VP1	mat_peptide	1992	2897	906	302	1799	2734	936	312
2A	mat_peptide	2898	3323	426	142	2735	3202	468	156
2B	mat_peptide	3324	3725	402	134	3203	3601	399	133
2C	mat_peptide	3726	4727	1002	334	3602	4612	1011	337
3A	mat_peptide	4728	5054	327	109	4613	4972	360	120
3B	mat_peptide	5055	5141	87	29	4973	5059	87	29
3C	mat_peptide	5142	5732	591	197	5060	5641	582	194
3D	mat_peptide	5733	7142	1410	469	5642	7048	1407	468
rhv like	Conserve domain	867	1238	372	124	566	1051	486	162
rhv like	Conserve domain	1506	1970	465	155	1274	1777	504	168
RNA_helicase	Conserve domain	4146	4445	300	100	4031	4330	300	100
Peptidase C3	Conserve domain	5142	5678	537	179	5060	5590	531	177
RNA_dep_RNAP	Conserve domain	6204	6962	759	253	6065	6868	804	268
3'UTR	3'UTR	7049	7096	48		7049	7096	48	

### Phylogenetic analysis of BPeV11 and BPeV20

According to BLASTx search, the ORF sequence of BPev11 and BPev20 shared the 50.44% and 50.47% identity at aa level with that of Ferret parechovirus (FPeV) isolate MpPeV1 (GenBank no. NC_034453) collected from *Mustela putorius furo*, respectively [3]. The 2C region of the BPev11 exhibited the greatest (60%) aa identity to FPeV, while its P1 and 3D displayed 46 and 56% identity to FPeV, respectively (Fig. 2B). For BPev20, the 3D region showed the highest (59%) aa identity to FPeV, while its P1 and 2C displayed 46 and 56% identity to FPeV, respectively (Fig. 2B).

**Fig. 3:**
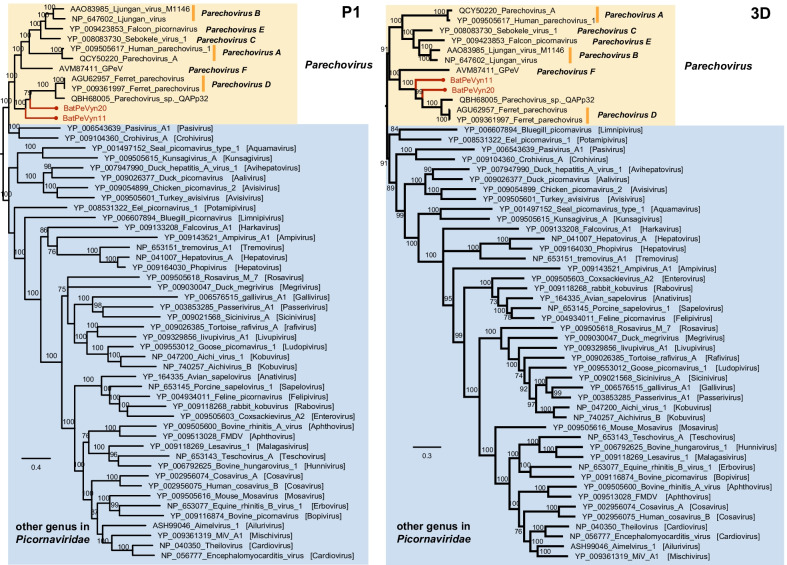
Phylogenetic tree of BPev11 and BPev20. Phylogenetic relationships between BPev11 and BPev20 and representative members of the genus *Parechovirus* and representative members of other genera in the family *Picornaviridae*, based on the predicted amino acid sequences of the different picornavirus coding regions: P1 (1211 aa) and 3D (566 aa). Scale bars indicate amino acid substitutions per site. BPev11 and BPev20 are labelled in red. Nodes with bootstrap values > 70 are noted

BPev11 and BPev20 were most closely to the genus *Parechovirus* of picornaviruses. Therefore, the representative members in the genus *Parechovirus* and other representative genera and species in *Picornaviridae* were selected as reference strains for the phylogenetic analysis. The phylogenetic relationships between BPev11 and BPev20 and the representative picornaviruses based on aa sequences of the different picornavirus coding regions (P1and 3CD) are shown in Fig. 3. Phylogenetic analysis of the P1 confirmed that BPev11 and BPev20 formed a monophyletic branch with the members within *Parechoviruses E* (two Ferret parechoviruses and an unclassified parechovirus)*,* while GPeV in *Parechovirus F* were also involved in this monophyletic clade based on the 2C and 3CD phylogenetic trees. However, BPev11 and BPev20 have distant relatedness to the viruses in same branch. According to the species demarcation criteria proposed by the International Committee on Taxonomy of Viruses (ICTV), the divergence (number of differences per site between sequences) between members of different Parechovirus species ranges from 0.44–0.63 for P1 and 0.34–0.59 for 3CD. This criterion suggests BPev11 and BPev20 are probably classified as representatives of a new species in the *Parechovirus* genus. Nevertheless, the members of species *Parechovirus D* and *Parechovirus F* relatively lack, the identification of new species needs further research.

## Discussion

With the rise of viral metagenomics analysis, new bat-borne viruses have been continuously discovered around the world, including influenza A virus [27], Phlebovirus [28], and Banyangvirus [29]. From the wide geographical locations of the various bat viruses detected so far, it is almost certain that we will see more and more disease outbreaks caused by bat viruses [14]. Bats harbour a more significant proportion of zoonotic viruses per host species than other mammalian orders [30, 31]. In some cases, outbreaks have been linked to bat roosting or foraging in close proximity to human settlements [32–34]. The driving factors for the increasing spillover events of bat viruses, particularly bat CoVs, are complex and most likely a combination of bat habitat disruption through climate change, increased urbanization pressure from humans, wildlife trade and animal markets [35, 36]. The SARS CoV outbreak in China, which caused more than 8000 cases of severe respiratory disease in humans resulting in 10% Mortality [37], was linked to Rhinolophus sp. bats and the wildlife trade [38]. Up to today, the virus members from families of *Rhabdoviridae*, *Orthomyxoviridae*, *Paramyxoviridae*, *Coronaviridae*, *Togaviridae*, *Flaviviridae*, *Bunyaviridae*, *Reoviridae*, *Arenaviridae*, *Herpesviridae*, *Picornaviridae*, *Hepesviridae* and *Adenoviridae*, have been isolated from different bat species [39], but no complete genome of Parechovirus has been found, only a 1080 nt parechovirus contig was recovered in a previous study [40]. As a natural reservoir, the bats deserve more studies to prevent the outbreaks of diseases caused by viruses.

This study collected 21 fecal samples of *H. armiger* from Mengla County of Xishuangbanna Prefecture, Yunnan province, to process metagenomic analysis, then acquired two nearly complete sequences BPev11 and BPev20. The isolation of BPev11 and BPev20 from *H. armiger* in China is the first complete genome of parechovirus isolations from bat feces of the genus *Hipposideros*. Nested-PCR was employed to investigate the prevalence of BPev11 and BPev20 using a primer pair. The positive rates of BPev11 and BPev20 both were 1/21. BPev11 showed an electrophoresis band in sample 11, while BPev20 showed an electrophoresis band in sample 20 (See Additional file 2). *Parechovirus* is a genus of RNA viruses with a poly(A) tail. Regrettably, after a series of trials, such as a 5′ Rapid Amplification for cDNA End (RACE) PCR and 3’ RACE, the 5’UTR and 3’UTR of BPev11 and BPev20 are not complete. Sequence identity matrices for P1, 2C, and 3D regions (Fig. 2B) and phylogenetic tree for P1 and 3D regions of BPev11 and BPev20 suggest BPev11 and BPev20 may be a novel species of genus *Parechovirus*. Although BPev11 and BPev20 are more distantly related to the viruses of *Parechovirus A* and *Parechovirus B*, further studying the host range restriction and pathogenicity is necessary.

## Conclusions

This study found two nearly complete genomes of parechoviruses, BPeV11 and BPeV20, in 21 fecal samples of *H. armiger* collected in Mengla County of Xishuangbanna Prefecture Yunnan Province, China. The prevalence of BPev11 and BPev20 by Nested-PCR showed that 1 of 21 fecal samples was positive. Based on amino acid identity comparison and phylogenetic analysis of P1, 2C, and 3D, BPeV11 and BPeV20 were closely related to but distinct from FPeVs. It was probably proposed to be a novel species in the genus *Parechovirus* of the family *Picornaviridae*. Until now, this is the first time that complete genome of parechovirus has been found in bat feces. However, further research is needed to uncover BPeV11 and BPeV20 in the pathogenic mechanism in animals and humans.

## Supplementary Information

**Additional file 1.** BPev11 and BPev20 MAFFT-alignment with other parechovirus polyproteins.

**Additional file 2.** The result of Nested PCR about BPev11 and BPev20.

## Data Availability

All genome sequences have been deposited into GenBank under accessions OK149219- OK149220. Quality-filtered sequence reads have been deposited in the sequence read archive (SRA) under the accession number SRR15885421.

## Abbreviations

NCBI: National Center for Biotechnology Information
ICTV: International Committee on Taxonomy of Viruses
NVNR: Non-virus non-redundant protein database
BLAST: Basic local alignment search tool
