# Supplementary material for: Identification and genome characterization of novel parechovirus sequences from Hipposideros armiger in China
Source: Virol J. 2022 May 15;19:80. doi: 10.1186/s12985-022-01806-1 (PMC9107582; doi:10.1186/s12985-022-01806-1)
Supplement: Supplementary file 1 — Additional file 1. BPev11 and BPev20 MAFFT-alignment with other parechovirus polyproteins. [file 12985_2022_1806_MOESM1_ESM.pdf]

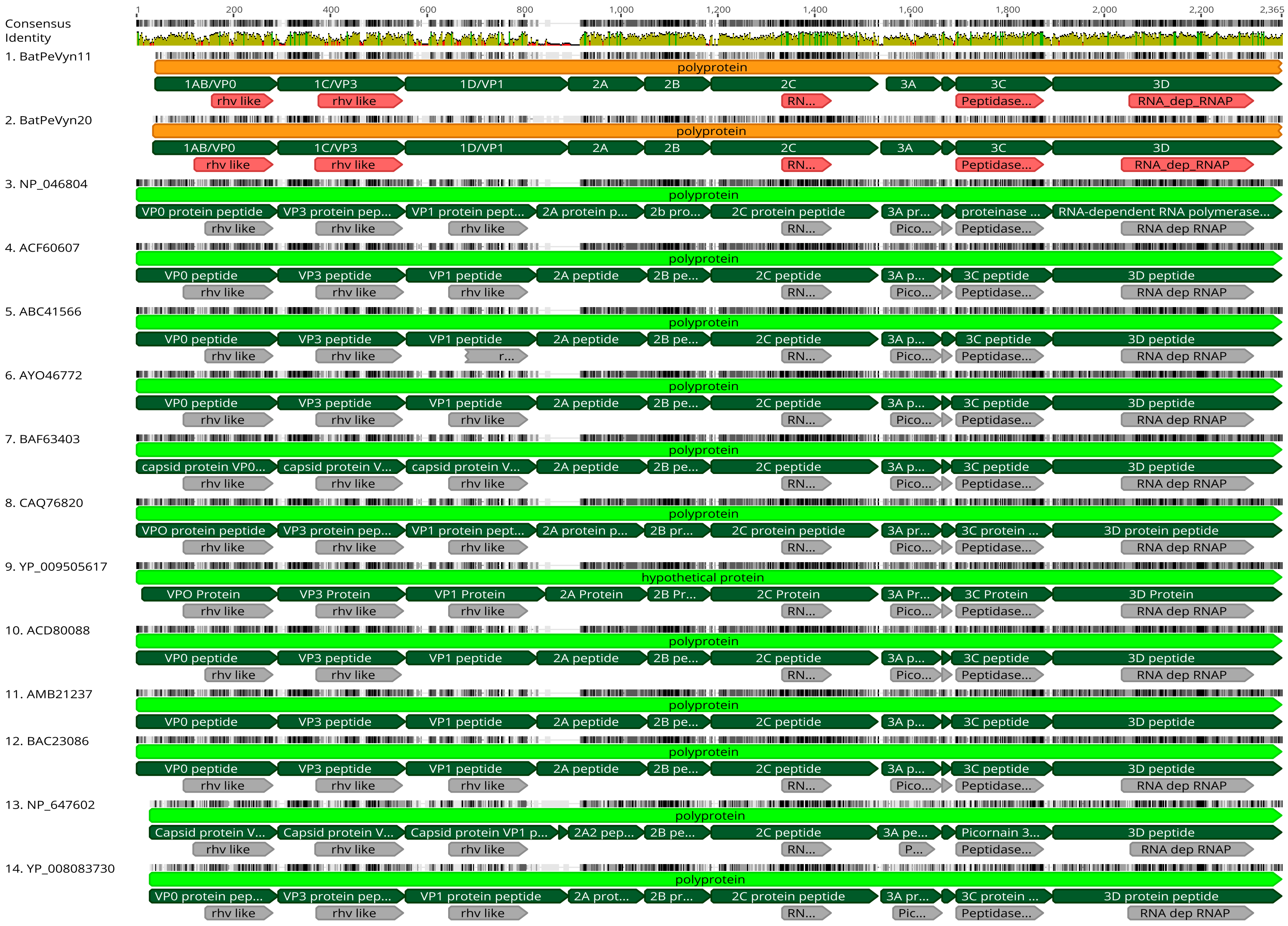

Additional file 1: BPev11 and BPev20 MAFFT-alignment with other parechovirus polyproteins.
A sequence identity graph is shown above the alignment, illustrating residue identity among sequences across all positions. Green represents complete amino acid identity for a given position, yellow represents less than total similarity, and red represents residues with very low similarity.
