# Supplementary figures and images for: Identification and genome characterization of novel parechovirus sequences from Hipposideros armiger in China
Source: Virol J. 2022 May 15;19:80. doi: 10.1186/s12985-022-01806-1 (PMC9107582; doi:10.1186/s12985-022-01806-1)

BPeV11

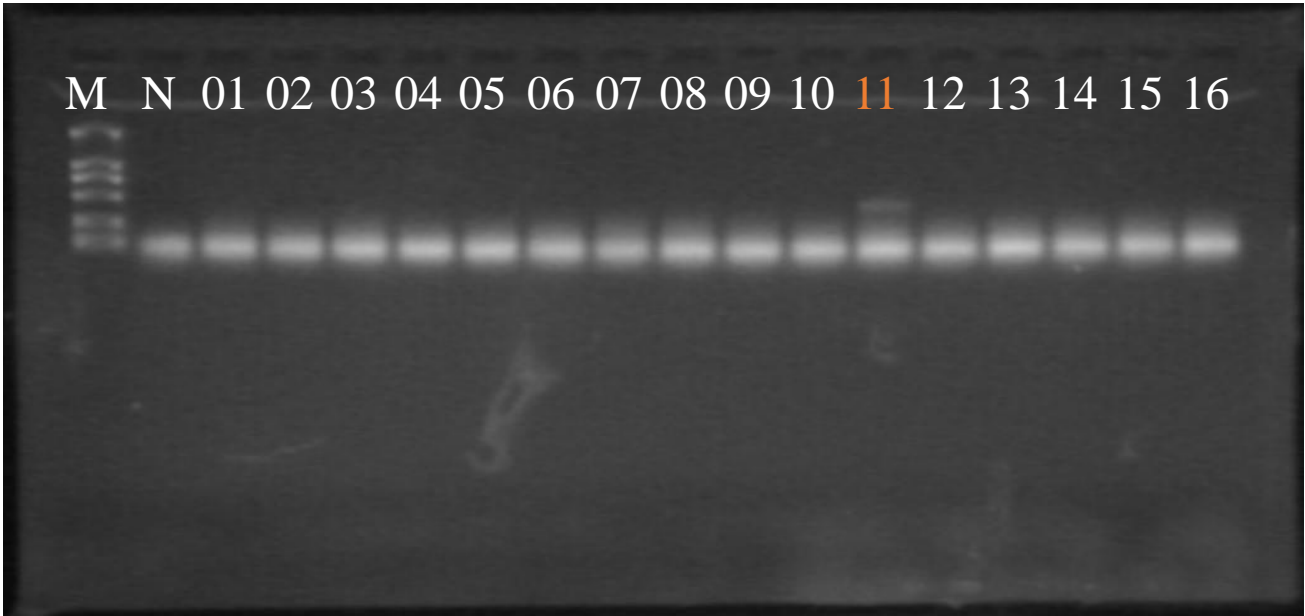

BPeV20

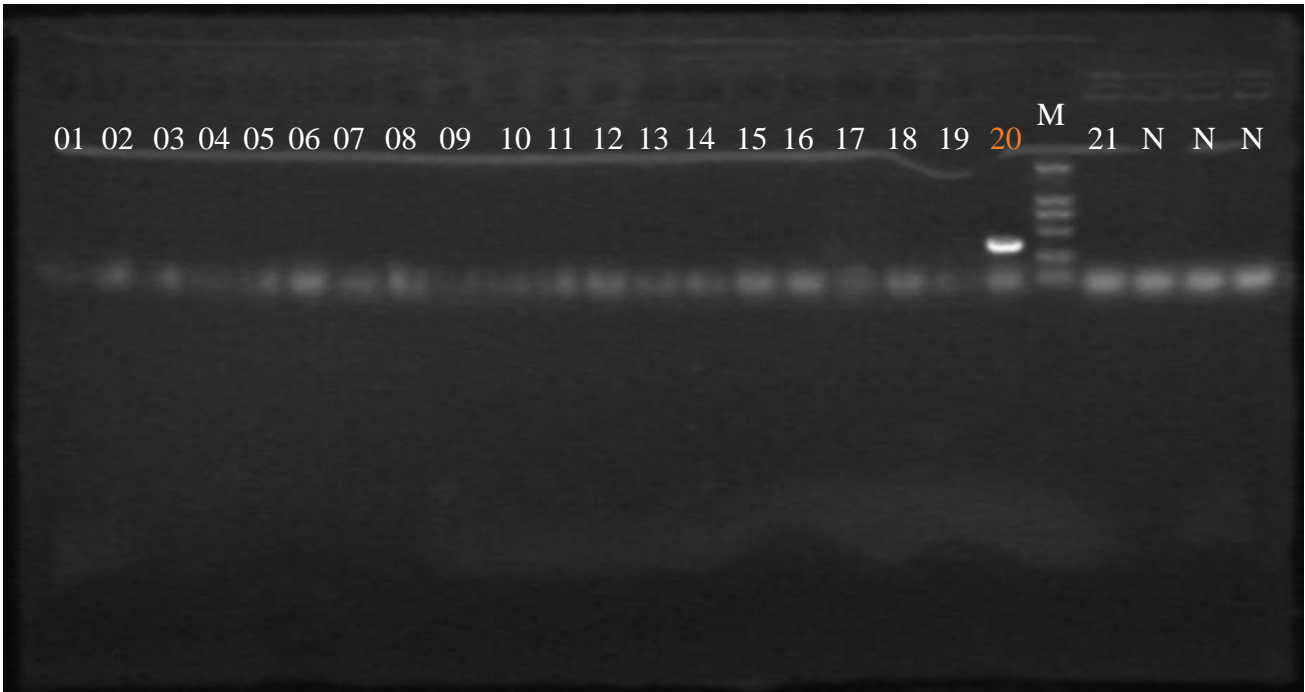

Supplement: Supplementary file 2 — Additional file 2. The result of Nested PCR about BPev11 and BPev20. [file 12985_2022_1806_MOESM2_ESM.pdf]
